# The comparison of grey-scale ultrasonic and clinical features of hepatoblastoma and hepatocellular carcinoma in children: a retrospective study for ten years

**DOI:** 10.1186/1471-230X-11-78

**Published:** 2011-06-25

**Authors:** Hua Zhuang, Yu-lan Peng, Tian-wu Chen, Yong Jiang, Yan Luo, Qiong Zhang, Zhi-gang Yang

**Affiliations:** 1 Department of Ultrasound, West China Hospital of Sichuan University, 37 Guo Xue Xiang, Chengdu, Sichuan 610041, China; 2 Department of Radiology, West China Hospital of Sichuan University, 37 Guo Xue Xiang, Chengdu, Sichuan 610041, China; 3 Department of Radiology, North Sichuan Medical College, 234 Fu Jiang Road, Nanchong, Sichuan 637007, China; 4 Department of Pathology, West China Hospital of Sichuan University, 37 Guo Xue Xiang, Chengdu, Sichuan 610041, China

## Abstract

**Background:**

Hepatoblastoma (HBL) and hepatocellular carcinoma (HCC) are respectively the first and the second most common pediatric malignant liver tumors. The purpose of this study was to evaluate the combined use of the ultrasound examination and the assessment of the patients' clinical features for differentiating HBL from HCC in children.

**Methods:**

Thirty cases of the confirmed HBL and 12 cases of the confirmed HCC in children under the age of 15 years were enrolled into our study. They were divided into the HBL group and the HCC group according to the histological types of the tumors. The ultrasonic features and the clinical manifestations of the two groups were retrospectively analyzed, with an emphasis on the following parameters: onset age, gender (male/female) ratio, positive epatitis-B-surface-antigen (HBV), alpha-fetoprotein increase, and echo features including septa, calcification and liquefaction within the tumors.

**Results:**

Compared with the children with HCC, the children with HBL had a significantly younger onset age (8.2 years vs. 3.9 years, P < 0.001) and a significantly smaller frequency of positive HBV (66.7% vs. 13.3%, P < 0.001). The septa and liquefaction were more frequently found in HBL than in HCC (25/30, 83.3% vs. 2/12, 16.7%, P < 0.001; 17/30, 56.7% vs. 3/12, 25%, P = 0.02). When a combination of the liquefaction, septa, negative HBV and onset age smaller than 5 years was used in the evaluation, the sensitivity was raised to 90%, the accuracy was raised to 88%, and the negative predictive value was raised to 73%.

**Conclusion:**

Ultrasonic features combined with clinical manifestations are valuable for differentiating HBL from HCC in children.

## Background

Two-thirds of the pediatric primary liver tumors are malignant [1,2]. Hepatoblastoma (HBL) accounts for 40-60% of the liver tumors in children, which is the most common malignant liver tumor in children [1,3]. Hepatocellular carcinoma (HCC) accounts for about 20%, which is the second most common malignant liver tumor in children [1,4]. The two malignant liver tumors have some similar clinical and imaging features but the prognosis and treatments are quite different. The origin and nature of the malignant liver tumors should be clear in order to use a proper treatment. The differential diagnosis should be based on the following findings: tumor encapsulation, calcification presence, current hemorrhage, predisposing factors including hepatic fibrosis, and some clinical information of the patients [5].

Typically, ultrasonography is the first-line imaging method of evaluating children with liver tumors. But few studies were performed to describe in detail the differentiation between the two types of the tumors, especially for description on the value of the combined use of the clinical manifestations and the ultrasound features for the differentiation [1-7]. Thus, the purpose of this study was to evaluate the ultrasound examination combined with the significant clinical manifestations of the patients in differential diagnosis between HBL and HCC in children.

## Methods

### Patients

This study was a retrospective one. Between January 1993 and May 2009, 42 children under 15 years old (mean age, 5.1 years; age range, 1-15 years) with the confirmed HBL or HCC were included into our study. They were divided into the HBL group (n = 30) and the HCC group (n = 12). The study was approved by the institutional review board of our hospital (registration number 20090622) and was in compliance with the Helsinki Declaration. All the children underwent abdominal ultrasound scanning before treatments. Among the patients, only 5 (1 with HBL, other 4 with HCC) underwent contrast CT. None of them underwent contrast MRI. The HBL group consisted of 13 girls and 17 boys ranging in age from 1 to 14 years, among which 12 were infected with hepatitis B virus (HBV). The HCC group consisted of 9 boys and 3 girls ranging in age from 6 to 15 years, among which 4 were infected with HBV. Only 5 children were tested for hepatitis C infection and all were anti-hepatitis-C-virus negative, including 3 children with HCC and 2 with HBL. The pathological diagnoses were obtained by liver biopsy, postoperative histological examinations or autopsy. Among the 42 patients, 25 underwent hepatic segmentectomy, lobectomy, trisegmentectomy or liver transplantation. Chemotherapy was given to 30 patients.

In the HBL group, according to the cellular pathology, 17, 6, 3 and 4 patients respectively had a tumor of the epithelial type, the mixed epithelial/mesenchymal type, the anaplastic type, and the macrotrabecular type.

### Ultrasonic image acquisition and analysis

The patients underwent the ultrasound scan with GE Logiq 500, HP SONOS 4500, or PHILIPS HD-11 color Doppler ultrasound diagnostic instruments. An experienced doctor, who had more than 5 years' work experience, examined the patients. The probe frequency of the ultrasound used in our study was 3-5 MHz. The patients were placed in the supine position, the gray-scale ultrasound of the lesion was used. The images were transferred to the workstation. A series of ultrasound features of the lesions (number, size, location, calcification, liquefaction, septa within the mass), together with the background liver echo nature, and the conditions of the portal hepatic venous systems were recorded.

Two experienced ultrasound specialists, respectively with 19 (Y. Peng) and 17(Y. Luo) years' work experience and were blinded to the diagnosis, reviewed the pictures and recorded a serial of ultrasound features as for the same contents mentioned above. Discrepancies in the interpretations between the specialists were resolved by the consensus.

The lesion numbers were recorded according to the distribution pattern of the solitary, multiple or diffuse lesions. To obtain the given size of the liver mass, the long-axe diameter was measured three times at the central section of the mass in the ultrasound images. The average diameter was calculated, which was regarded as the final diameter. The location of the lesion was recorded as the left, right or bi-lobe of the liver. Liquefaction, calcification and septa were observed in the grey-scale ultrasound images. The diagnosis of liver cirrhosis was made if a small and/or nodular liver along with increased echogenicity and an irregular appearing was found. The echo nature of the background liver was recorded as hepatic cirrhosis or not.

### Statistical analysis

We performed data analysis on a personal computer with the SPSS statistical package (version 13.0 for Windows, SPSS Inc., Chicago, IL, USA). Statistical analyses (t-test, Wilcoxon's rank sum test, Chi-Square test or Fisher exact test) were performed to determine the differences in the onset age, male/female ratio, hepatitis-B-surface-antigen (HBS-Ag), alpha-fetoprotein (AFP) increase, abdominal pain, palpable abdominal lump, jaundice, lesion size, lesion number, and echo nature (septa, liquefaction, calcification) within the lesions between the two groups. A *P *value of less than 0.05 was considered statistically significant.

## Results

### Clinical manifestations

**Figure 1:**
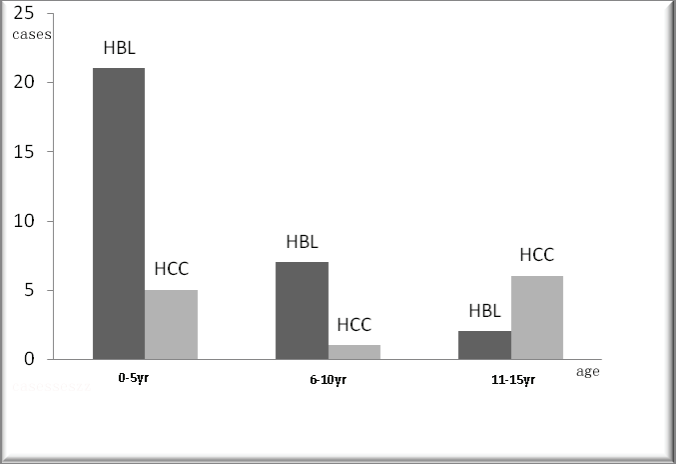
**Histogram shows the different distributions of hepatoblastoma (HBL) and hepatocellular carcinoma (HCC) related to the onset age**.

The clinical manifestations of the patients were compared between the HCC and HBL groups (Table 1). There was no significant difference in the gender ratio (male/female), jaundice, palpable abdominal lump, AFP increase or abdominal pain (*P *> 0.05). Both the onset age and the positive HBS-Ag were significantly different between the two groups (*P *< 0.001). In the histogram for the onset age of the patients in the HBL group and the HCC group, a different distribution could be observed (Figure 1). The onset age was younger than 5 years in 21 patients of the HBL group, whereas older than 10 years in 6 children of the HCC group. That is to say, the average tumor onset-age was younger and the negative HBS-Ag appeared more frequently in the children with HBL than in the children with HCC.

**Table 1:** Clinical manifestations of HBL and HCC

Clinical manifestation	HBL (*n *= 30)	HCC (*n *= 12)	*P*
**Onset age (yrs)**	3.9 ± 2.8	8.2 ± 3.1	< 0.001
Gender ratio*	2.8:1 (22/8)	3:1 (9/3)	> 0.05
**Palpable abdominal lump**	8/30 (26.7%)	4/12 (33%)	> 0.05
**Abdominal pain**	11/30 (36.7%)	4/12 (33%)	> 0.05
**AFP increase**	17/30 (56.7%)	7/12 (58.3%)	> 0.05
**HBS-Ag positive**	4/30 (13.3%)	8/12 (66.7%)	< 0.001
**Jaundice**	17/30 (56.7%)	5/12 (41.7%)	> 0.05

HBL: hepatoblatoma; HCC: hepatocellular carcinoma * refers to the male to female ratio

### Ultrasonic features

**Figure 2:**
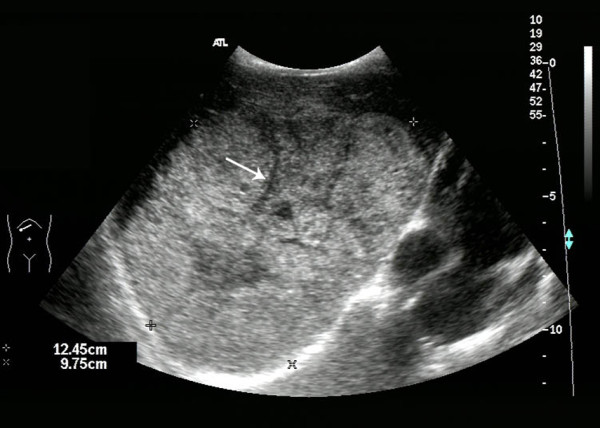
**HBL in the right lobe of the liver in a 2-year-old girl**. A grey scale ultrasound image revealed the septa within the lesions (arrow).

**Figure 3:**
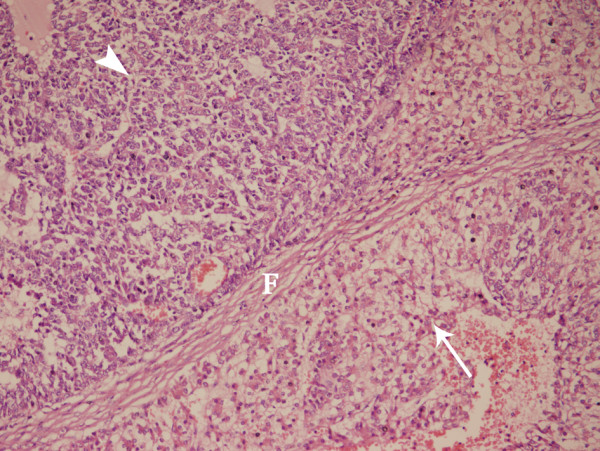
**HBL in the right lobe of the liver in a 2-year-old girl**. Photomicrograph (original magnification, 180×; hematoxylin-eosin stain) revealed a fibrous band (F) between the fetal (arrow) and the embryonic (arrow head) type of the HBL cells.

**Figure 4:**
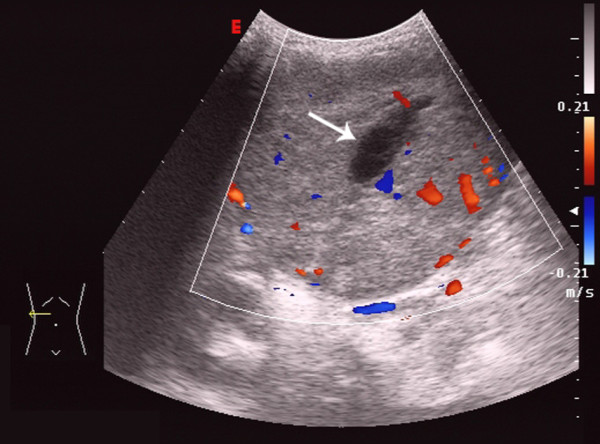
**HBL in the right lobe of the liver in a 4-year-old boy**. The liquefaction (arrow) was observed in the center of the tumor and some blood signals were also observed.

**Figure 5:**
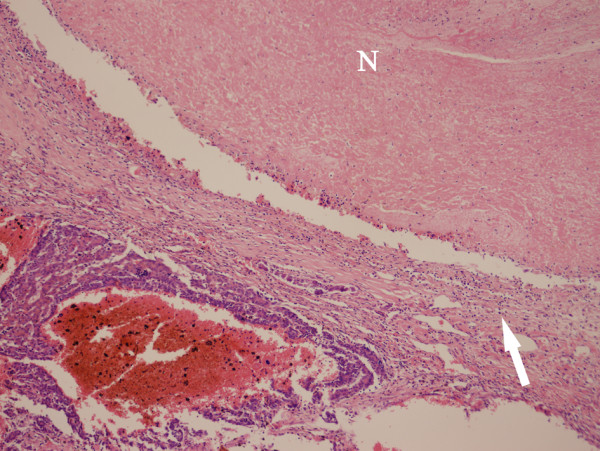
**HBL in the right lobe of the liver in a 4-year-old boy**. Photomicrograph (original magnification, 90×; hematoxylin-eosin stain) demonstrated some fibrous bands (arrow) and necrosis (N) within the tumor.

**Figure 6:**
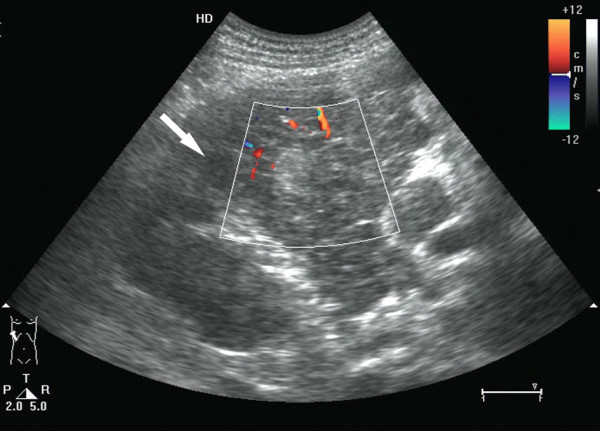
**HCC in the right lobe of the liver in a 10-year-old boy with hepatic cirrhosis after the HBV infection**. A diffusely-distributed heterogeneous mass (arrow) with no septa or liquefaction was revealed at a setting of cirrhosis.

The ultrasonic features of HCC and of HBL were listed in Table 2. There was no significant difference in the location or the number of the tumor between the two groups (*P *> 0.05). The echo within the tumor mass was significantly different in the septa and the liquefaction between the two diseases (*P *< 0.001, *P *= 0.02 respectively). That is to say, septa and liquefaction were easier to be found in HBL than in HCC (Figure 2, 3, 4, 5). But the calcification was occasionally found in both the two kinds of tumors. The lesion size was significantly different between the two groups (*P *< 0.001). The long-axe diameters of HBL and HCC were 8.5 ± 3.5 cm and 3.7 ± 1.8 cm, respectively. There was no significant difference in the bile duct, the vessel involvement or the blood flow condition (*P *> 0.05). The portal vein thrombosis and the hepatic vein thrombosis were observed in a few cases of HCC. The background liver cirrhosis was observed only in the children with HCC (Figure 6).

**Table 2:** Ultrasonic features of HBL and HCC

	HBL (*n *= 30)	HCC (*n *= 12)	*P*
**Lesion number**			
**Solitary**	22/30 (73.3%)	8/12 (66.7%)	> 0.05
**Multiple**	5/30 (16.7%)	3/12 (25%)	
**Diffuse**	3/30 (10%)	1/12 (8.3%)	
**Lesion location**			
**Left liver**	9/30 (30%)	3/12 (25%)	> 0.05
**Right liver**	13/30 (43.3%)	5/12 (41.7%)	
**Bi-lobe**	8/30 (26.7%)	4/12 (33.3%)	
**Lesion echo**			
**Liquefaction**	17/30 (56.7%)	3/12 (25%)	= 0.02
**Calcification**	4/30 (13.3%)	2/12 (16.7%)	> 0.05
**Septa**	25/30 (83.3%)	2/12 (16.7%)	< 0.001
**Lesion size**	8.5 ± 3.5 cm	3.7 ± 1.8 cm	< 0.001
**Blood vessel involvement**			
**PVT**	0/30 (0%)	2/12 (17%)	> 0.05
**HVT**	0/30 (0%)	1/12 (8.3%)	> 0.05
**Liver cirrhosis**	0/30 (0%)	4/12 (33.3%)	< 0.001

PVT: portal vein thrombosis; HVT: hepatic vein thrombosis

### Ultrasonic features combined clinical manifestations for diagnosis

Based on the ultrasonic features and the clinical manifestations, the statistics was made to analyze the diagnosis of HBL (Table 3). When a single parameter was used, e.g., when the negative HBS-Ag was used, sensitivity of 87% (26/30), specificity of 83% (10/12), and accuracy of 83% (35/42) were obtained; when the septa was used, the positive predictive value (PPV) of 93% (25/27) and the negative predictive value (NPV) of 67% (10/15) were obtained; when the negative HBS-Ag was used, NPV of 67% (8/12) was obtained. However, when a combination of the liquefaction, septa, onset age (< 5 years) and negative HBS-Ag was used, the sensitivity was raised to 90%, the accuracy was raised to 88% and NPV was raised to 73%.

**Table 3:** Significant ultrasonic and clinical findings for HBL

Ultrasonic & clinical finding	Sensitivity (%)	Specificity (%)	Accuracy (%)	PPV (%)	NPV (%)
**Liquefaction (1)**	57(17/30)	75 (9/12)	62 (26/42)	85(17/20)	41 (9/22)
**Septa (2)**	83 (25/30)	83 (10/12)	83(35/42)	93(25/27)	67(10/15)
**Onset age** **< 5 yrs(3)**	70 (21/30)	58 (7/12)	67(28/42)	81(21/26)	44(6/16)
**HBS-Ag negative (4)**	87 (26/30)	67 (8/12)	81(34/42)	80(24/30)	67(8/12)
**(1)+(2)+(3)+(4)**	90 (27/30)	83 (10/12)	88(37/42)	93(27/34)	73(8/11)

Numbers in parentheses: case number; PPV: positive predictive value;

NPV: negative predictive value; yrs: years

## Discussion

Liver tumors are rare in children. Primary liver tumors account for approximately 1% of the tumors in children. About 50-60% of the primary liver tumors in children are malignant, and more than 65% of those malignant liver tumors are HBL [1-4]. The constant annual incidence of HBL in children is 0.5-1.5 per 1 million in Western countries [6]. The currently accepted hypothesis is that the HBL cells are derived from the pluripotent hepatic stem cells. Those stem cells retain the ability to differentiate into both the hepatocytes and the billiary epithelial cells, and retain the ability to express markers for both the cell types as a feature in HBL [7]. HCC is the second most common pediatric primary malignant liver tumors, accounting for about 20% of the primary liver tumors in children [1,4]. The two diseases can be treated by chemotherapy and surgical resection, or by liver transplantation as a last resort [8,9]. It seems that children with HBL have a better survival rate when compared with children with HCC [3]. Recently, approximately 75% of children with HBL can be cured completely [7].

The results of this study have indicated that children with HCC have a higher incidence of the positive HBV than those with HBL. Many studies have revealed that HBV is related to HCC in children. HBV has been considered a major etiological factor of HCC in children beyond the age of 4 years [10]. However, little is known about the etiology of HBL. The most well-established risk factors of HBL are Beckwith-Wiedemann syndrome, family history of familial adenomatous polyposis, low birth weight, and smoking by either or both of the parents [7]. This may explain the different incidence of the HBV infection related to HBL and HCC in children in this study.

HBL was mainly a tumor occurring in early childhood [6,7], and 30-50% of the HBL cases occurred in the first year of childhood and 50-90% before the age of 5 years [2,3,6,7]. The onset age of HCC was older than that of HBL in children. Lee, et al. reported that there was an HCC onset peak at 12 years old [10]. In our series, the average HBL onset-age was 2.9 years, but the average HCC onset-age was 9.3 years. Frequently, HCC was one of the long-term results of chronic viral infection [11]. And 80% of the HCC cases developed in the cirrhotic livers [12]. In developing countries, young patients were apt to suffer from chronic hepatitis B virus infection [13,14]. The time from liver cirrhosis to HCC might be the main reason for the older onset-age of HCC. In our study, positive HBV was found in 66.7% of the HCC cases but in 13.3% of the HBL cases. These findings have supported the idea that HBV is a major etiological factor of HCC in children beyond the age of 4 years.

There are more boys than girls who suffered from HBL or HCC, which was one of the common characteristics of those two types of tumors occurring in children [15]. The results of our study have also supported this finding. The imaging study is important in evaluation liver neoplasms. CT, MRI and ultrasound are the most-commonly-used modalities for pediatric doctors in their medical researches as well as their clinical practice. Ultrasound is accepted as a first-line imaging method because of its less irradiation, greater convenience and better real-time [16]. Ultrasound is extremely valuable in detecting much smaller lesions, especially in detecting fluid and blood-flow in a lesion, and it also can evaluate the hepatic vascular anatomy [17]. As a rule, the initial diagnosis of live tumor is usually made by the abdominal ultrasound examination. Complete surgical resection is a key to the permanent cure of the disease, and so ultrasound examination can be used to exactly localize the tumor and assess the extent of the tumor development. Scintigraphy with 99mTc-labelled monoclonal anti-AFP is mainly used in the tumor staging. However, the clinical usefulness of this technique for HBL is not completely clear [16].

The lesion sizes were significantly different between the two groups. The long-axe diameter of HBL was greater than that of HCC. The younger onset-age of the tumor was a possible reason. The children with HBL could not tell exactly about their early symptoms, so the diagnosis was made relatively late. Although the average long-axe diameter of HBL was greater than that of HCC, there were some overlaps between the two groups. Therefore, the lesion size could not be used as an important indicator for differentiating HBL from HCC.

The two types of tumors in this study had some similarities in the ultrasound features. Firstly, most of the lesions in both HBL and HCC groups were located in the right lobe of the liver in more than 40% of the cases. Our study showed that the location of HBL was similar to that reported in the previous studies, in which the tumor location was in the right lobe of the liver in 60-70% of the HBL cases [7,12]. Secondly, most of the patients with HBL or HCC had solitary lesions (66.7% in the HCC group, 73.3% in the HBL group), which coincided with what the medical literature had reported [12]. However, these two diseases had many different imaging features. Dachman, et al. reported that liquefaction might be caused by intralesional necrosis or hemorrhage [12]. Typically, ultrasound could identify liquefaction as a hypoechoic area at the center of the lesion. In our series, liquefaction appeared in 25% of the HCC cases but appeared in 56.7% of the HBL cases; therefore, a significant difference could be observed between the two groups. So, liquefaction could be considered significant for the HBL diagnosis.

Calcification occurred in such liver diseases as HBL, HCC, teratoma and involuting haemangioma. So, calcification was not a specific indicator in the differential diagnosis [17]. Calcification might cause acoustic shadowing, which was better depicted by CT or ultrasound rather than MRI [8]. Microscopically, the calcification presence on radiographs was often associated with the osteoid presence [11]. Histologically, calcification was often related to the mixed type of HBL, and the reason was probably that there was a formation of the osteoid foci [12]. Calcification rarely occurres in the HCC patients, which is related to the radiation therapy [13]. Jha, et al. [2] reported that calcification was observed in about 40% of the HCC cases and observed in 50% of the HBL cases [2,8]. Our study showed that calcification was occasionally found in the two kinds of tumors that had not been treated before. But our study still failed to give any evidence that calcification is a specific indicator in the imaging studies for a differentiation of HBL from HCC. The reason for the great difference in calcification between the two groups might be that there were quite a few cases of the mixed epithelial/mesenchymal type of tumors.

Septa were formed by the fibrous tissue band around or inside the lesions. Histologically, HBL is usually present as a solitary large solid mass, which could contain fibrous bands, leading to a "spoked-wheel appearance" [2]. The typical fibrous septa could be observed in the grey-scale ultrasound images in our study. A previous case-study suggested that in a multifocal setting, the presence of a dominant lobulated mass was a clue to the diagnosis of HBL [8]. In our series, the septa were more commonly observed in untreated HBL than in HCC. Thus, this ultrasound feature can be used as an indicator for differentiating HBL from HCC. Among the ultrasonic and clinical features of the HBL lesions, the negative HBS-Ag was the most sensitive parameter, and the septa were the most specific parameter. When a combination of liquefaction, septa, onset age (< 5 years) and negative HBS-Ag was used, the sensitivity was raised to 90%, the accuracy was raised to 88%, and the negative predictive value was raised to 73%.

Our study still had some limitations including a long duration (1993-2009) of the materials collection. Besides, at the beginning of the study, the Color Doppler Flow Imaging (CDFI) and elastography were not introduced into the practice. Whereas contrast enhanced ultrasound (CEUS) has already been mature in the diagnosis of the liver occupying lesions in recent years, childhood is still a contraindication to the contrast agent SonoVue. Thus, we were unable to give enough information about the CDFI, CEUS and elastography manifestation of HBL and HCC in children. But we think that our finding is still valuable for the pediatric physicians.

## Conclusion

In summary, HBL is more likely to develop in children younger than 5 years, with negative HBS-Ag and ultrasonic manifestation of a mass that has septa and liquefaction. HCC usually develops in older children with an HBV infection, with a liver solid mass, which has no liquefaction or septa. Ultrasound is the first-line imaging method to be used in a child with a suspected liver tumor, and this imaging method can provide information to help decide the use of the clinical treatment. Ultrasonic features combined with clinical manifestations are valuable for differentiating HBL from HCC in children.

## Competing interests

The authors declare that they have no competing interests.

## Authors' contributions

HZ and ZGY designed the research; YL and YLP did the ultrasound examination; YJ and QZ collected the data; HZ and TWC drafted and revised the manuscript and approved the final version; HZ wrote the first draft of this paper. All authors contributed to the intellectual content. All authors read and approved the final manuscript.

## Pre-publication history

The pre-publication history for this paper can be accessed here:

http://www.biomedcentral.com/1471-230X/11/78/prepub
